# Electromyography Analysis of the Masseter Muscle's Activity in the Management of Oral Submucous Fibrosis

**DOI:** 10.7759/cureus.59675

**Published:** 2024-05-05

**Authors:** Poornachitra P, Arvind Muthukrishnan, Suresh Venugopalan, Ruwan D Jayasinghe, Vaishnavi Rajaraman, Uma Maheswari T N

**Affiliations:** 1 Department of Oral Medicine and Radiology, Saveetha Dental College, Saveetha Institute of Medical and Technical Sciences, Saveetha University, Chennai, IND; 2 Department of Prosthodontics and Implantology, Saveetha Dental College, Saveetha Institute of Medical and Technical sciences, Saveetha University, Chennai, IND; 3 Faculty of Dental Sciences, University of Peradeniya, Peradeniya, LKA; 4 Department of Prosthodontics and Implantology, Saveetha Dental College, Saveetha Institute of Medical and Technical Sciences, Saveetha University, Chennai, IND

**Keywords:** premalignant lesions, masseter muscle, emg, electromyography, osf, osmf, oral submucous fibrosis

## Abstract

Introduction

Oral submucous fibrosis (OSMF) is a persistent, collagen metabolic disorder distinguished by the presence of fibrosis of the connective tissue stroma in the oral mucosa with a higher malignant potential rate for oral cancer. This study aimed to analyze the utility of electromyography (EMG) as the prognostic assessment tool in the management of OSMF with conventional intralesional corticosteroid therapy.

Materials and methods

This study included 20 OSMF cases of age range 20 to 80 years without systemic comorbidities to assess pre-treatment and post-treatment changes with intralesional corticosteroid therapy as an intervention and to determine if it could be assessed using electromyographic study. Clinical and histopathological grading of OSMF was done. The five clinical parameters were evaluated for measuring treatment prognosis. Among them, mouth opening, tongue protrusion, and burning sensation assessments were quantitative parameters, and palpable fibrotic bands and mucosa colour were qualitative parameters. As OSMF involves changes in muscle plane in moderately advanced and advanced cases, EMG was used as an assessment tool for measuring muscle activity. Among the muscles of mastication, the masseter and temporalis were selected for evaluation. Twenty age and gender-matched healthy controls were required for this study as there are no standardized normal values for amplitude and onset of activity in muscle analysis. The EMG activity of the right and left temporalis and masseter muscles were recorded using surface electrodes and were correlated with five clinical assessment parameters.

Results

In the right masseter, the rest amplitude of 1.6010 µV of the OSMF was statistically significant (p-value: 0.050) when compared with 4.1275 µV of the control. The clench amplitude of 133.370 µV of the OSMF was statistically significant (p-value: 0.062) when compared with 94.310 µV of the control. In the left masseter, the rest amplitude of 1.6695 µV of the OSMF was statistically significant (p-value 0.066) when compared with 2.5735 µV of the control. In the left masseter, the onset of muscle action of 62.670 ms of the OSMF was statistically significant (p-value: 0.017) when compared with 131.835 ms of the control. The clench amplitude differences in the right masseter of 133.370 µV pre-treatment, and 102.775 µV post-treatment were statistically significant (p-value: 0.007). The clench amplitude in the left masseter of 102.535 µV pre-treatment, and 92.090 µV post-treatment were statistically significant (p-value: 0.036). The correlation was seen between tongue protrusion and rest amplitude in the right masseter in OSMF (r = 0.376, p-value: 0.023).

Conclusion

There was a correlation between tongue protrusion and rest amplitude in the right masseter muscle in OSMF patients before treatment. In the right and left masseter, during rest, the amplitude of the OSMF group was lesser than that of the control group. During clench, in the right masseter, the amplitude of the OSMF group was higher than that of the control group. During clench in the left masseter, the onset of muscle action was lesser in the OSMF group than in the control group. After treatment, there was a reduction in clench amplitude in OSMF patients from their pretreatment values signifying muscle relaxation and a better onset of muscle action.

## Introduction

Oral submucous fibrosis (OSMF) is defined as a chronic, insidious disease that affects parts of the oral cavity and at times the pharynx. Although occasionally preceded and/or associated with vesicle formation, it is always associated with juxta‑epithelial inflammatory reaction followed by fibro‑elastic changes in lamina propria with epithelial atrophy leading to stiffness of the oral cavity [1]. OSMF was defined by Pindborg in 1966 and the current widely accepted definition is based on his description [2]. It was also previously called by other names, namely, idiopathic scleroderma of mouth, idiopathic palatal fibrosis, sclerosing stomatitis, and juxta-epithelial fibrosis [3-5]. OSMF is now one of the well-recognised potentially premalignant oral epithelial lesions (PPOELs) [6].

OSMF is commonly seen in Southern and Southeast Asian region, including India [7]. The prevalence is 2.01% and the rate of transformation to malignancy is 2.3-7.6%. In the Indian subcontinent, instability in the genome and altered keratinocyte phenotype had been proved for its role into malignant transformation [7-12]. The aetiology of OSMF is betel nut/areca nut chewing in a regular manner for a long duration. The alkaloids present in areca nut are responsible for the pathological effects. Among the alkaloids present, namely, arecoline, arecaidine, guvacine and guvacoline, arecoline undergoes a process called nitrosation. This results in the formation of N-nitrosoguvacoline, N-nitrosoguvacine, and 3-methyl nitroso aminopropionitrile [13], which causes alkylation of DNA leading to the formation of cyanoethyl that binds with 6-O-methylguanine. Prolonged exposure to 6-O-methylguanine irritant leads to malignant transformation [5]. Also, consistent daily chewing of areca nuts leads to frictional tissue alterations followed by chronic inflammation and the release of fibrogenic cytokines [14].

The clinical features of OSMF include a burning sensation in the oral mucosa, ulceration and pain, reduced jaw movement, depapillation of the tongue, blanching, and leathery texture with loss of pigmentation of oral mucosa leading to progressive restriction of mouth opening. The common sites are buccal mucosa, labial mucosa, and soft palate. The affected tissue will be symmetrical and progresses to firm nature and paleness. The common complaint in OSMF patients is a progressive stiffness of the cheeks that restricts mouth opening [14]. There also exist restrictions in tongue protrusion in OSMF patients that get improved throughout treatment. 

Binne and Cawson [15] using light microscopy proved that OSMF fibrils are thinner and smaller in diameter than normal fibrils. There is the involvement of the muscle plane in OSMF pathogenesis with masseter muscle hypertrophy observed due to constant overloading of the jaws due to excess masticatory force in crushing areca nut products. Therefore, the fibrils per unit area are increased than in normal musculature. Degenerating muscle fibres are seen in the collagenous sub-epithelial zone. Labban and Canniff [16] had shown using electron microscopy that severe degenerative changes happen in a high proportion of muscle fibres. Fibres have large pools of oedematous fluid-like homogenous material between muscle cells.

Muscle action is characterized by contraction force (amplitude) and the onset of activity in microvolts and milliseconds, respectively. They are measured using electromyography (EMG), which could be surface or needle type. Surface EMG (sEMG) is commonly preferred because of its non-invasiveness and ease of measurement. In the orofacial region; the common muscles measured are masseter, temporalis, buccinator, orbicularis oris, digastric, and sternocleidomastoid, which gets activated during gnathic movements. However, in OSMF patients, the muscles of mastication to be studied are the masseter, temporalis, medial, and lateral pterygoid. Since lateral and medial pterygoids can be measured only using invasive needle EMG, it is commonly not included in sEMG.

Bio-EMG (Bio-research Associates, Inc., Milwaukee, Wisconsin, United States) activity on masseter muscle has also been studied extensively in neurological diseases and in temporomandibular joint disorders. There were EMG studies in assessing muscle involvement in OSMF patients [17-19] in which non-invasive sEMG had been used to measure the activity of the masseter muscle for quantifying the treatment outcome. However, till date, there has been no study that has quantified the EMG activity range in oral submucous fibrosis in relation to healthy comparators. The reason for this lacuna might be the concentrated prevalence of OSMF in developing countries with the cultural habit of betel nut or areca nut chewing. 

Though the early involvement of the masseter muscle in OSMF and its changes after the treatment had been studied with EMG, the diagnostic and prognostic quantification of muscle activity change is yet to be researched. Whether the change in muscle activity influences OSMF management with intralesional corticosteroid therapy or whether intralesional corticosteroid is effective in altering muscle activity has to be researched. Additionally, an increase in EMG amplitude could be a consistent finding in chronic tobacco product chewers, which also needs to be studied. 

This study was designed to understand the effectiveness of treatment outcomes in the management of OSMF with intervention. The aim of this study was to compare and analyze the utility of EMG as the prognostic assessment tool in the management of OSMF with conventional intralesional corticosteroid therapy in a private dental hospital setup. The objective was to evaluate the effectiveness of intralesional corticosteroid therapy in managing OSMF and the effectiveness of EMG in assessing the treatment outcome.

## Materials and methods

This was a prospective, quasi-experimental clinical study, which was approved by the Scientific Review Board and Institutional Human Ethical Committee of Saveetha Dental College and Hospitals (SRB/SDC/OMED-2002/21/TH-024; IHEC/SDC/OMED-2002/21/TH-014). The trial was also registered with the Clinical Trial Registry of India (CTRI/2021/10/037650). The sample size was based on the statistical evaluation of the previous study [18]. With a power of 90% and a 10% loss to follow-up, the total case sample size was approximated to 20 using G*Power software (Heinrich Heine University Düsseldorf, Düsseldorf, Germany). 

The patients who reported to the Department of Oral Medicine and Radiology of Saveetha Dental College and Hospitals, Chennai, India, with a chief complaint of restricted mouth opening and burning sensation, and who also had a history of smokeless tobacco use were screened. Twenty clinically diagnosed OSMF cases without any systemic comorbidities were recruited for this study in the case group. Twenty age- and gender-matched healthy comparators without systematic comorbidities were selected for the control group. The study also included the assessment of the effectiveness of EMG in evaluating the treatment outcome. As there were no standard reference values in Bio-EMG, age- and gender-matched individuals were used as comparators in evaluating the correlation between OSMF patients and healthy individuals. The patients with systemic comorbidities, patients with a history of OSMF treatment who had not followed up treatment care and those who required surgical management, patients <18 years of age, patients with temporomandibular joint (TMJ) disorders, patients with orofacial asymmetry, patients with orofacial manifestations of syndromes, and completely edentulous patients were excluded from this study.

The selected 20 clinically diagnosed OSMF patients of the age range 20 to 80 years without systemic comorbidities were subjected to incisional biopsy in the area of the quid holding site with the most taut fibrotic bands after obtaining informed consent from the patients. The grading followed for OSMF was based on Kerr et al.'s classification (Table 1) [6], which is widely accepted for its conglomeration of clinical presentation, functional mouth opening, and histopathological analysis when compared to other numerous classifications reported across the literature. Grade 5 OSMF cases were not included in our data collection as suspected oral squamous cell carcinoma changes when detected were subjected to oncology care for biopsy and further management without delay in treatment care. 

**Table 1:** Kerr et al.'s classification of oral submucous fibrosis [6]

Grade	Description
Grade 1	Mild. Any features of oral submucous fibrosis disease triad and interincisal opening of more than 35mm.
Grade 2	Moderate. The above features of oral submucous fibrosis with interincisal opening limited to 20-35mm.
Grade 3	Severe. Clinical features of oral submucous fibrosis with an interincisal opening less than 20 mm.
Grade 4A	Oral submucous fibrosis with other features of the oral potentially malignant disorder.
Grade 4B	Oral submucous fibrosis with any grade of epithelial dysplasia on biopsy.
Grade 5	Oral submucous fibrosis with oral squamous cell carcinoma.

**Figure 1:**
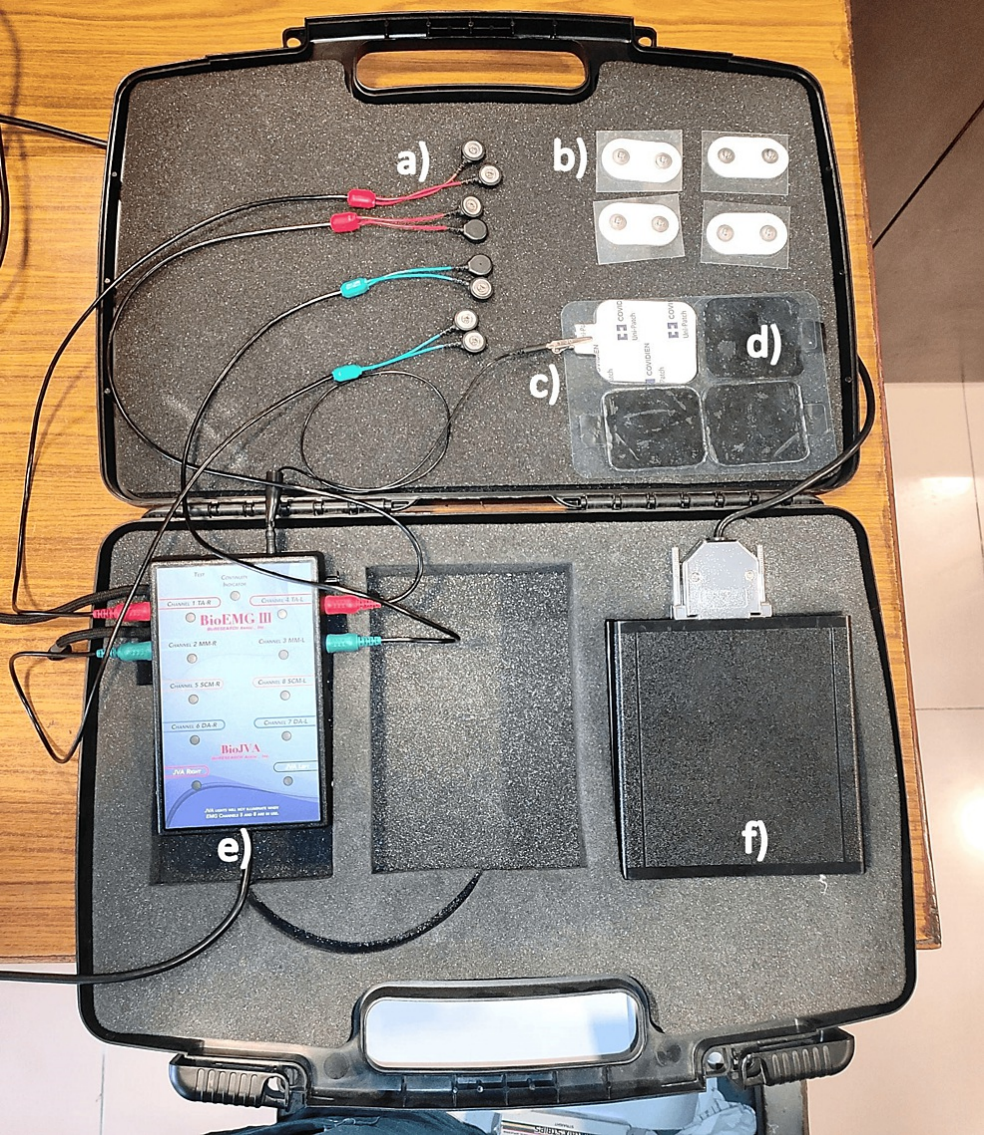
Bio-EMG* a) masseter and temporalis electrode wires b) disposable wet gel electrodes c) ground electrode clip d) surface ground electrode e) continuity indicator f) microcontroller *Bio-research Associates, Inc., Milwaukee, Wisconsin, United States

The EMG armamentarium used for the present study included a measuring scale; Bio-EMG equipment (Figure 1) consisting of masseter and temporalis electrode wires, disposable wet gel electrodes, a ground electrode clip, a surface ground electrode, a continuity indicator, and a microcontroller; a laptop with licensed BioPak analysis software (DigitalDental, Paddington, New South Wales, Australia); uninterrupted power supply; and controlled air-conditioned room devoid of external noises. The muscles assessed using Bio-EMG were the masseter and temporalis before and after treatment by objective characterization.

**Figure 2:**
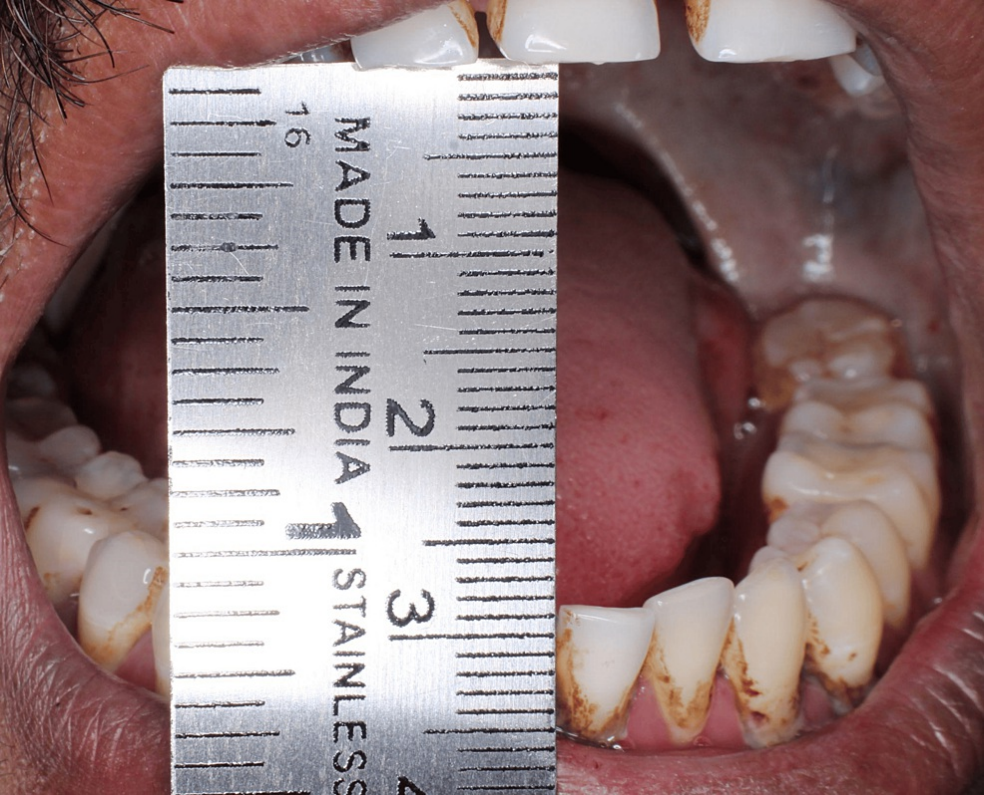
Measuring mouth opening (in mm)

**Figure 3:**
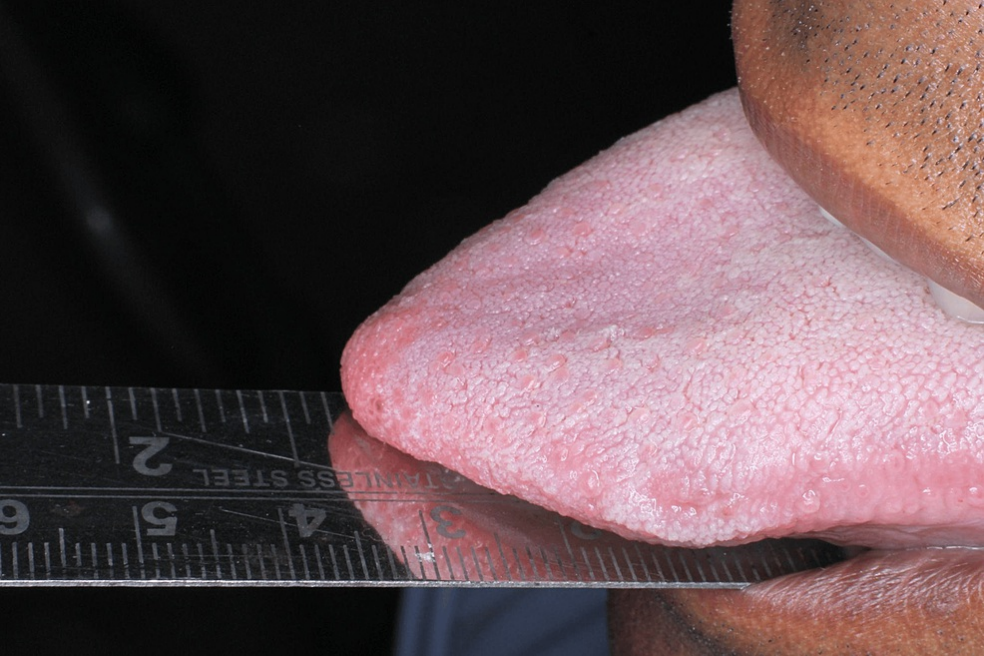
Measuring tongue protrusion (in mm)

**Figure 4:**
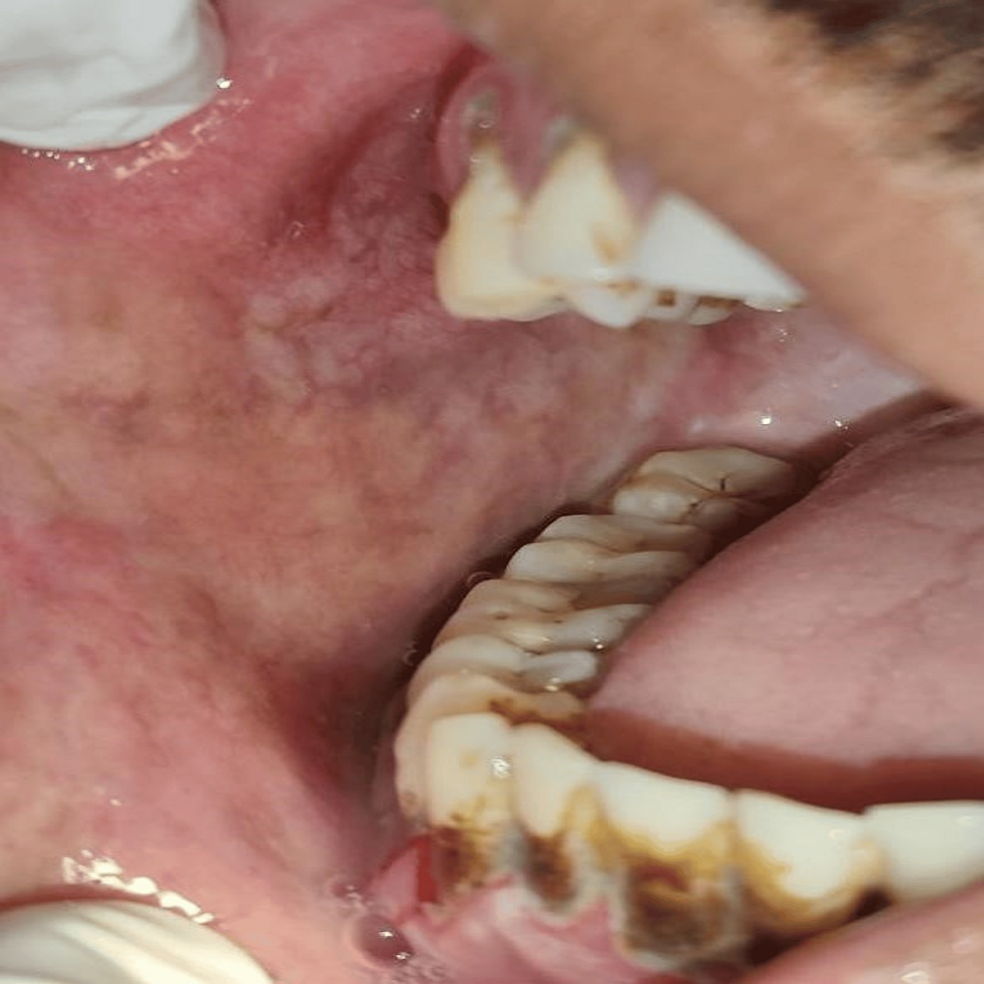
Vertical fibrotic bands

**Figure 5:**
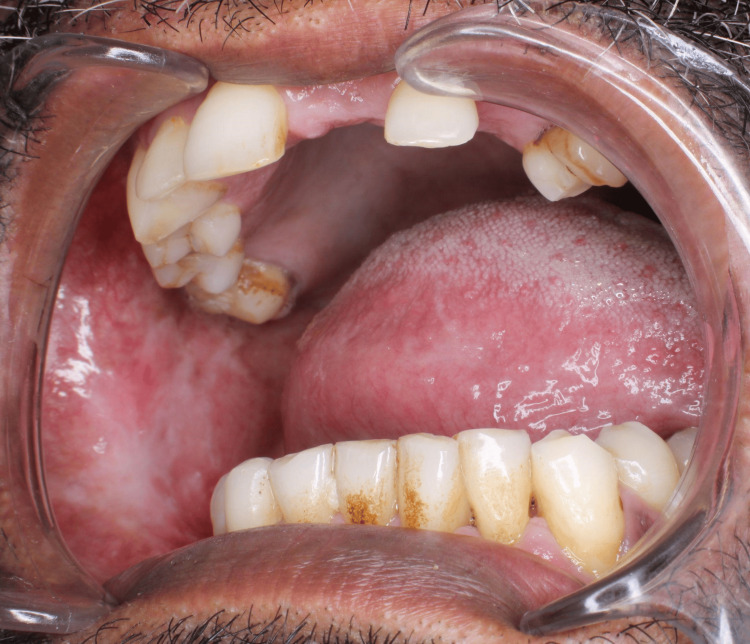
Pale, blanched mucosa

The five clinical assessment parameters recorded for OSMF patients were mouth opening, tongue protrusion, palpable fibrotic bands, the colour of the mucosa, and burning sensation. Mouth opening was measured in millimetres using a measuring scale from the incisal edge of the upper anterior to lower anterior teeth (Figure 2). Tongue protrusion was measured in millimetres using a measuring scale from the incisal edge of the lower anterior teeth to the tip of the protruded tongue (Figure 3). Palpable fibrotic bands ranged from bilateral to unilateral presentation on the buccal mucosa (Figure 4). Mucosa colour ranged from pale, blanched mucosa in OSMF patients to normal pink or light pink in healthy individuals (Figure 5). The burning sensation was measured using the Visual Analogue Scale (VAS) scoring ranging from 0 to 10.

The OSMF cases were subjected to EMG assessment before treatment using Bio-EMG at rest and during clench. The recording was done in a controlled noiseless room at 17 degrees Celsius room temperature by a single trained operator with no active electronic devices nearby while recording to prevent signal cross interference. The patient was made to sit in a comfortable upright posture in a natural head position with the Frankfort horizontal plane (FHP) parallel to the floor. The skin was prepared using 70 percent alcohol surface disinfectant to avoid impedance due to oil secretions. The exploring wet gel electrodes at a spacing of 3-4 mm were made to stick on the prepared surface at the site of most taut muscle fibre bilaterally on the temporalis and masseter muscles, and the ground electrode was stuck on the shoulder muscle for conduction circuit closure. The exploring electrodes were positioned on the thickest part of the muscle parallel to the muscle fibre orientation. This site was confirmed with clinical palpation aided by functional assessment.

**Figure 6:**
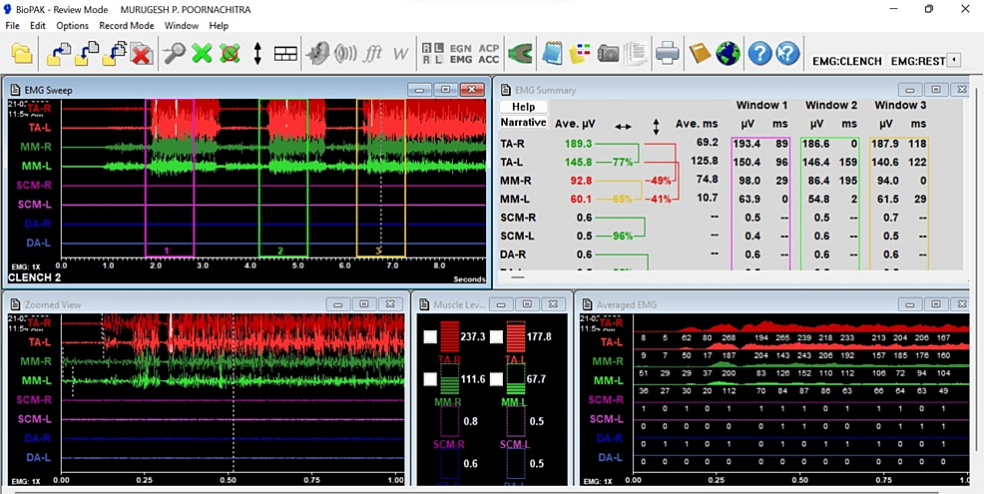
Sample image of recorded Bio-EMG* of an OSMF patient OSMF: oral submucous fibrosis *Bio-research Associates, Inc., Milwaukee, Wisconsin, United States

The electrode wires were inserted into the conducting circuit box, which was relayed through a microcontroller that was attached to the laptop with BioPak Software. The recorded signals were amplified using a differential amplifier, with a fixed gain of 5,000, common-mode rejection ratio (hardware only) > 130 dB at 60 Hertz, signal-to-noise ratio of 1,000,000 to 1, common-mode voltage range -3.0 to +3.0 volts DC, and bandwidth 30 to 1000 Hertz (@ 2,000 Hz sample rate). The EMG software displays the signals as original time domain windows and the average of three levels that disclose contraction patterns and relative intensities (Figure 6). The muscle activity at rest was recorded first followed by muscle activity during teeth clenching. The simultaneous bilateral activity was assessed in both the temporalis and masseter muscles. Resting EMG measured only the electrical activity during rest. The contraction force was described as amplitude in microvolt units and the onset of muscle activity during clenching was measured in milliseconds.

The pre-treatment EMG was followed by anti-tobacco cessation counselling and a pharmacotherapy regimen of 12 doses of intralesional corticosteroid therapy that comprised hyaluronidase 1500 IU and dexamethasone sodium acetate 4 mg/ml admixed with 0.5 ml of 2% lignocaine, which was given twice a week at regular intervals. The patient was also advised lycopene 5000 mcg antioxidant supplement once a day along with jaw stretching physiotherapy exercises. After 12 doses, post-treatment EMG was measured similarly to pre-treatment EMG in a controlled environment.

The EMG values and the five clinical parameters of OSMF were recorded before and after the intervention. Twenty age and gender matched healthy controls without systemic illness were selected and EMG activity of the temporalis and masseter muscles was recorded. All the readings were documented, tabulated, and formatted in Microsoft Excel (Microsoft Corporation, Redmond, Washington, United States) for statistical analysis. The primary outcome of this study was aimed to assess the EMG activity of the masticatory muscle in OSMF and in healthy individuals and also to compare the pre- and post-treatment muscle activity in OSMF. The secondary outcome was to assess the changes in the mouth opening, palpable fibrotic bands, the colour of the mucosa, burning sensation, and tongue protrusion of patients during the course of treatment.

The statistical analyses were comprehensively done using IBM SPSS Statistics for Windows, Version 26, (Released 2019; IBM Corp., Armonk, New York, United States). The numerical values were represented in mean and standard deviation (SD) with a 95% confidence interval of the difference. The numerical values deviated from the normal distribution and the two-tailed significance was greater than 0.05. Hence the continuous outcome variables of OSMF patients and healthy controls were determined to be highly skewed which was, therefore, further analyzed using non-parametric (NPr) methods. The descriptive statistics of frequency distribution were used to analyze the gender distribution, quid-held site, and OSMF grading within groups. The descriptive analysis was also used to assess the five clinical parameters before and after treatment.

Correlation analyses were done between the quantitative clinical assessment parameters of mouth opening (in mm), tongue protrusion (in mm), burning sensation scoring (0-10), and EMG values, and the correlation coefficient range (r) from -1 to +1 was considered for measuring the strength of linear association. The Mann-Whitney U test was used to compare the differences between the pre-treatment rest and clench EMG values of temporalis and masseter muscles of both OSMF and control groups, which were independent of each other. The Wilcoxon signed-rank test was used to compare the differences between the pre-treatment and post-treatment muscle activity EMG values of the OSMF group before and after the intervention. Both temporalis and masseter muscle activity at rest and clench were analyzed. A p-value ≤ 0.05 was considered statistically significant.

## Results

**Figure 7:**
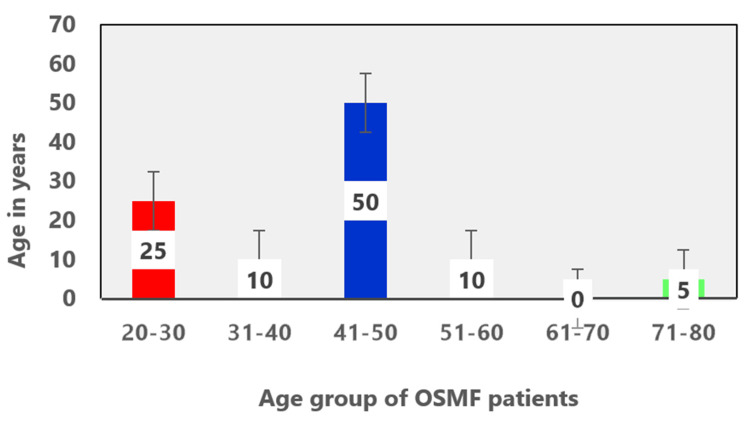
Frequency distribution of age of oral submucous fibrosis (OSMF) patients

The 20 OSMF cases (N=20) were grouped into six groups according to age range. A total of 25% (five) were in the 20-30 age range, 10% (two) were in the 31-40 age range, 50% (10) were in the 41-50 age range, 10% (two) were in the 51-60 age range, and 5% (one) were in 71-80 age range (Figure 7). The predominant age group of OSMF cases was 41-50 years. The 20 age and gender-matched individuals were also from this same age group with statistically accepted differences of plus or minus two years of age.

Group statistics with a two-tailed significance test showed that there was no statistically significant difference between the OSMF and the control groups (Table 2). The crosstabulation reporting and Pearson's Chi-square test were used to analyze the relationship between gender and groups and were statistically not significant with a p-value of 1.000 (Table 3).

**Table 2:** Group statistics with two-tailed significance

	Group	N	Mean	Standard deviation	Significance (two-tailed)
Age	Oral submucous fibrosis	20	42.90	12.502	0.921
	Control	20	42.50	12.788	

**Table 3:** Gender group crosstabulation: Pearson's Chi-square test asymptotic significance (two-sided): 1.000

			Group		
			OSMF	Control	Total
Gender	Male	Count	16	16	32
		% within group	80.0%	80.0%	80.0%
	Female	Count	4	4	8
		% within group	20.0%	20.0%	20.0%
Total		Count	20	20	40
		% within group	100.0%	100.0%	100.0%

The clinical parameters of OSMF cases assessed before the commencement of the treatment were tabulated (Table 4). A total of 65% of cases held the tobacco quid with areca nut products on the right side and 35% of cases on the left side of the oral cavity. All 20 cases had bilateral palpable fibrotic bands with pale, blanched oral mucosa. In clinical grading, 55% of OSMF cases were grade 4B, 35% were grade 2, and 10% were grade 3. Among histopathological grading done using Khanna and Andrade's classification, 60% were grade 4B, 25% were grade 4A, and 15% were grade 3 [20].

**Table 4:** Frequency distribution of pre-treatment parameters in oral submucous fibrosis

Pre-treatment parameters		Frequency	Percent
Quid held site	Right	13	65.0
	Left	7	35.0
	Total	20	100.0
Kerr et al.'s oral submucous fibrosis grading [6]	2	7	35.0
	3	2	10.0
	4B	11	55.0
	Total	20	100.0
Khanna and Andrade's oral submucous fibrosis grading [20]	3	3	15.0
	4A	5	25.0
	4B	12	60.0
	Total	20	100.0
Palpable fibrotic bands	Right and left	20	100.0
Mucosa colour	Pale, blanched	20	100.0

**Figure 8:**
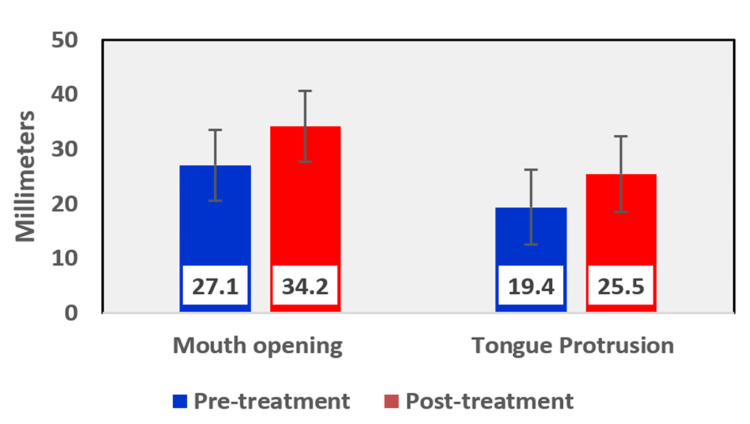
Graphical representation of improvement in mouth opening and tongue protrusion before and after treatment

The mean values of pre-treatment and post-treatment assessment parameters in the management of oral submucous fibrosis patients are tabulated in Table 5. All cases had bilateral palpable fibrotic bands that were reduced to a unilateral single band corresponding to the quid holding site (Table 4). The colour of the mucosa became normal pink after treatment from a pale blanched appearance (Table 4) due to improved vasculature. The burning sensation of oral mucosa resolved completely to score zero in all 20 cases after intralesional corticosteroid therapy. The mean increase in mouth opening was from 27.10 mm to 34.20 mm and the mean increase in tongue protrusion was from 19.40 mm to 25.50 mm respectively (Table 5, Figure 8).

**Table 5:** Descriptive statistics for correlation of oral submucous fibrosis parameters before and after treatment VAS: Visual Analogue Scale

Parameter	Stage of treatment	Mean	Standard deviation
Mouth opening (in mm)	Pre-treatment	27.10	6.504
	Post-treatment	34.20	6.494
Tongue protrusion (in mm)	Pre-treatment	19.40	6.840
	Post-treatment	25.50	6.902
Burning sensation (VAS score)	Pre-treatment	8.00	.000
	Post-treatment	0.00	.000
Rest (amplitude) in microvolts - right temporalis	Pre-treatment	2.3550	1.45299
	Post-treatment	1.8935	.98863
Clench (amplitude) in microvolts - right temporalis	Pre-treatment	118.9010	71.58737
	Post-treatment	117.975	85.0830
Clench (onset of muscle activity) in milliseconds - right temporalis	Pre-treatment	48.125	55.8866
	Post-treatment	83.315	116.8445
Rest (amplitude) in microvolts - left temporalis	Pre-treatment	2.2015	1.44434
	Post-treatment	1.9520	.82830
Clench (amplitude) in microvolts - left temporalis	Pre-treatment	111.305	52.1386
	Post-treatment	99.325	60.4084
Clench (onset of muscle activity) in milliseconds - left temporalis	Pre-treatment	52.890	54.2847
	Post-treatment	107.945	124.2779
Rest (amplitude) in microvolts - right masseter	Pre-treatment	1.6010	.65912
	Post-treatment	1.5240	.93676
Clench (amplitude) in microvolts - right masseter	Pre-treatment	133.370	67.5507
	Post-treatment	102.775	59.7803
Clench (onset of muscle activity) in milliseconds - right masseter	Pre-treatment	54.355	77.4924
	Post-treatment	68.855	86.3501
Rest (amplitude) in microvolts - left masseter	Pre-treatment	1.6695	1.10577
	Post-treatment	1.5125	1.19608
Clench (amplitude) in microvolts - left masseter	Pre-treatment	102.535	53.5130
	Post-treatment	92.090	59.2750
Clench (onset of muscle activity) in milliseconds - left masseter	Pre-treatment	62.670	63.4549
	Post-treatment	78.345	76.2581

**Figure 9:**
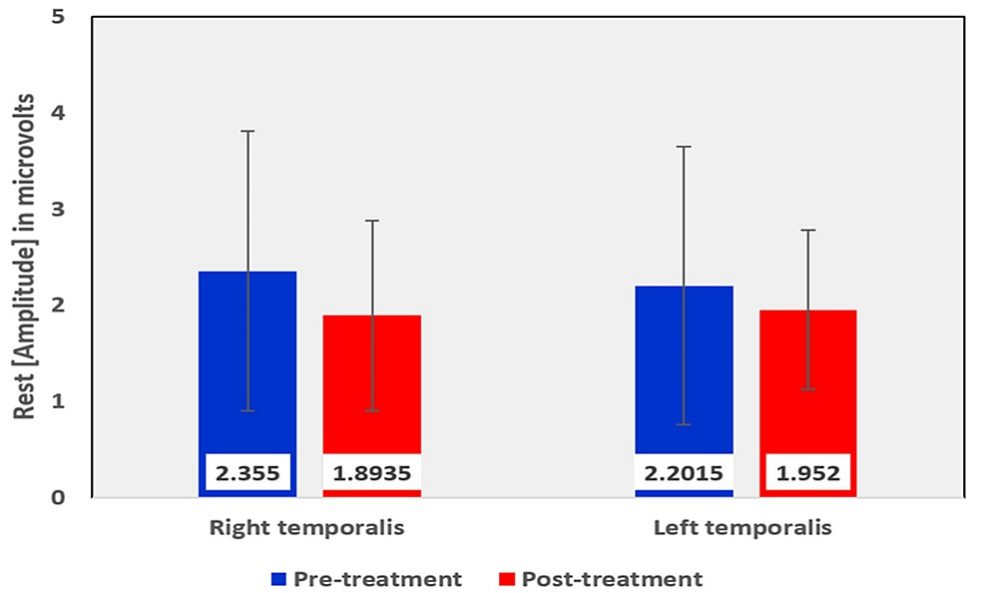
Graphical representation of the amplitude of the temporalis muscle at rest before and after treatment

**Figure 10:**
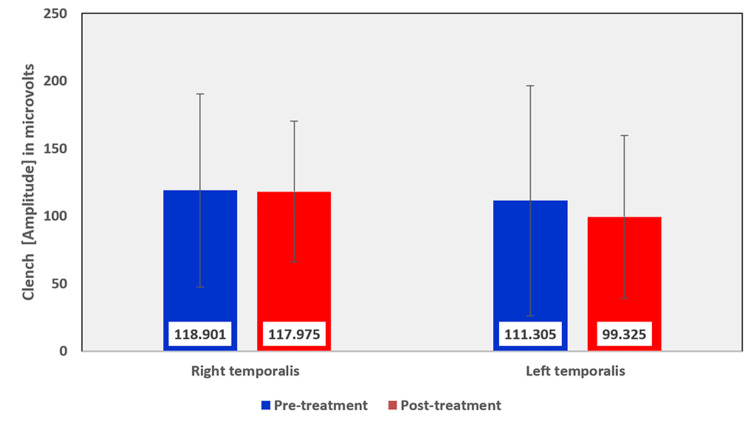
Graphical representation of the amplitude of the temporalis muscle during clench before and after treatment

**Figure 11:**
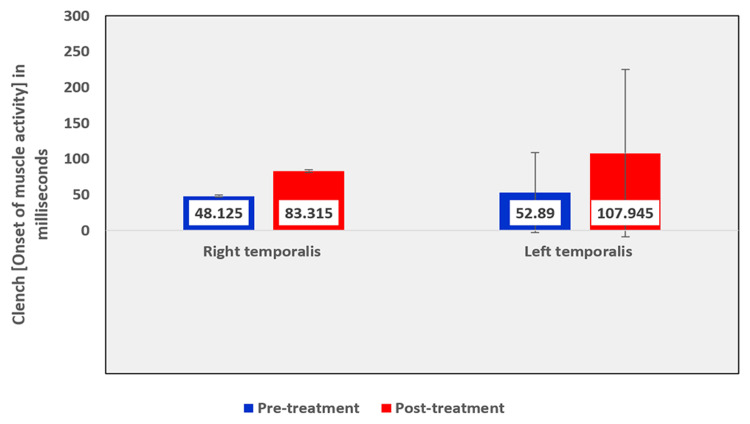
Graphical representation of the onset of muscle activity of the temporalis muscle during clench before and after treatment

Among the EMG assessment parameters of the temporalis muscle, when comparing the pre and post-treatment values in the OSMF group, the mean rest amplitude of right and left temporalis reduced from 2.355 µV to 1.8935 µV and 2.2015 µV to 1.952 µV, respectively (Table 5, Figure 9). The mean clench amplitude of right and left temporalis reduced from 118.901 µV to 117.975 µV and 111.305 µV to 99.325 µV, respectively (Table 5, Figure 10). The mean onset of muscle activity during clenching in the right and left temporalis increased from 48.125 ms to 83.315 ms and 52.89 ms to 107.945 ms, respectively (Table 5, Figure 11).

**Figure 12:**
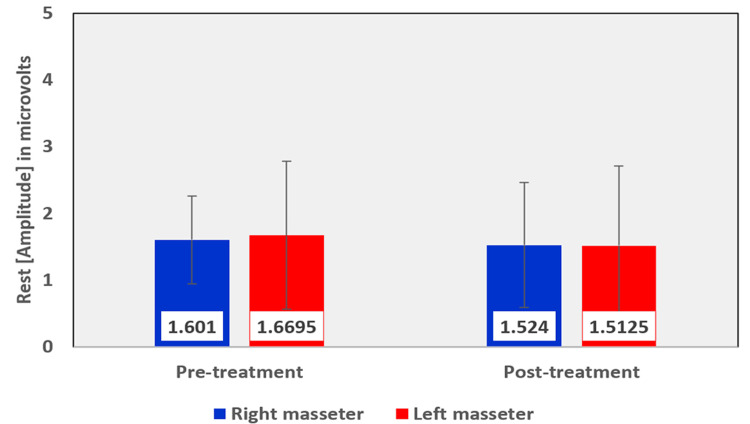
Graphical representation of the amplitude of the masseter muscle at rest before and after treatment

**Figure 13:**
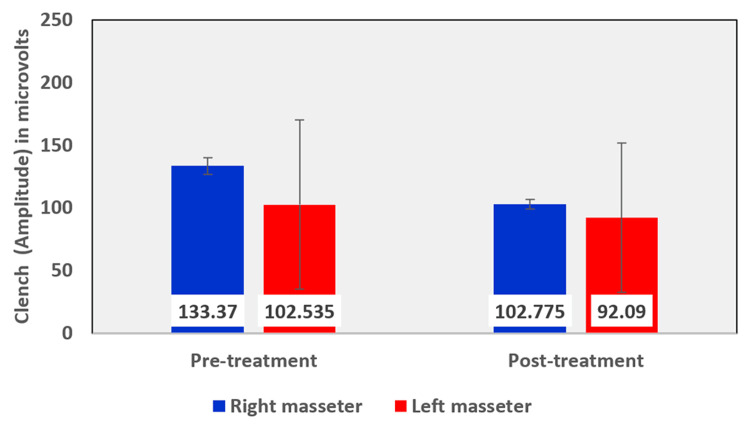
Graphical representation of the amplitude of the masseter muscle during clench before and after treatment

**Figure 14:**
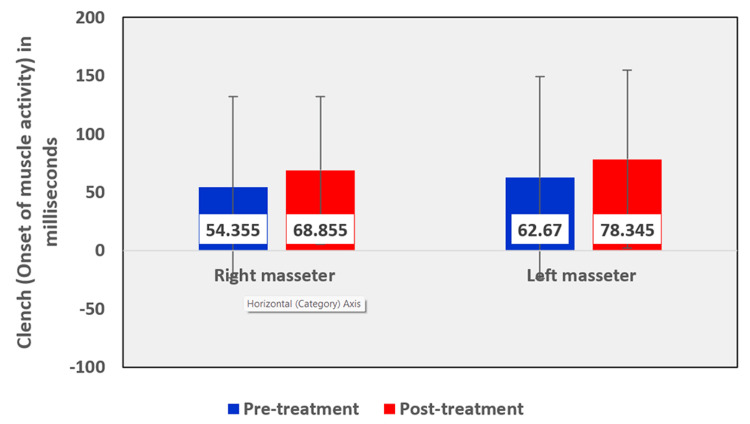
Graphical representation of the onset of muscle activity of the masseter muscle during clench before and after treatment

Among the EMG assessment parameters of the masseter muscle, when comparing the pre- and post-treatment values in the OSMF group, the mean rest amplitude of the right and left masseter reduced from 1.6010 µV to 1.5240 µV and 1.6695 µV to 1.5125 µV, respectively (Table 5, Figure 12). The mean clench amplitude of the right and left masseter reduced from 133.370 µV to 102.775 µV and 102.535 µV to 92.090 µV respectively (Table 5, Figure 13). The mean onset of muscle activity during clenching in the right and left masseter increased from 54.355 ms to 68.855 ms and 62.670 ms to 78.345 ms, respectively (Table 5, Figure 14).

The Mann-Whitney U test was used to compare the differences between the pre-treatment rest and clench EMG values of temporalis and masseter muscles of both OSMF and control groups on both the right and left sides (Table 6). The differences in amplitude at rest in the right masseter of the OSMF group, with a mean value of 1.6010 µV were statistically significant with a p-value of 0.050 when compared with the same of the control group, with the mean value of 4.1275 µV. The differences in amplitude during clench in the right masseter of the OSMF group, with a mean value of 133.370 µV were statistically significant with a p-value of 0.062 when compared with the same of the control group, with a mean value of 94.310 µV. The differences in amplitude during rest in the left masseter of the OSMF group, with a mean value of 1.6695 µV were statistically significant with a p-value of 0.066 when compared with the same of the control group, with the mean value of 2.5735 µV. The differences in onset of muscle action (duration) during clench in the left masseter of the OSMF group, with a mean value of 62.670 ms was statistically significant with a p-value of 0.017 when compared with the same of the control group, with a mean value of 131.835 ms. The differences between pre-treatment rest, and clench EMG values of the right and left temporalis muscles of both OSMF and control groups were not statistically significant.

**Table 6:** Mann-Whitney U test comparing the mean differences of pre-treatment rest amplitude, clench amplitude, and the onset of activity during clenching in the right and left temporalis and masseter muscles

	Group	N	Mean	Standard deviation	Asymptotic significance (two-tailed)
Rest (amplitude) in microvolts - right temporalis - pre-treatment	Oral submucous fibrosis	20	2.3550	1.45299	.766
	Control	20	2.8790	2.64682	
Clench (amplitude) in microvolts - right temporalis - pre-treatment	Oral submucous fibrosis	20	118.9010	71.58737	.433
	Control	20	92.9500	55.32892	
Clench (onset of muscle activity) in milliseconds - right temporalis - pre-treatment	Oral submucous fibrosis	20	48.125	55.8866	.935
	Control	20	40.730	47.1929	
Rest (amplitude) in microvolts - left temporalis - pre-treatment	Oral submucous fibrosis	20	2.2015	1.44434	.598
	Control	20	4.1835	7.25915	
Clench (amplitude) in microvolts - left temporalis - pre-treatment	Oral submucous fibrosis	20	111.305	52.1386	.465
	Control	20	99.445	60.7541	
Clench (onset of muscle activity) in milliseconds - left temporalis - pre-treatment	Oral submucous fibrosis	20	52.890	54.2847	.464
	Control	20	57.645	78.5048	
Rest (amplitude) in microvolts - right masseter - pre-treatment	Oral submucous fibrosis	20	1.6010	.65912	.050
	Control	20	4.1275	8.47137	
Clench (amplitude) in microvolts - right masseter - pre-treatment	Oral submucous fibrosis	20	133.370	67.5507	.062
	Control	20	94.310	57.3731	
Clench (onset of muscle activity) in milliseconds - right masseter - pre-treatment	Oral submucous fibrosis	20	54.355	77.4924	.123
	Control	20	76.990	69.8054	
Rest (amplitude) in microvolts - left masseter - pre-treatment	Oral submucous fibrosis	20	1.6695	1.10577	.066
	Control	20	2.5735	2.93534	
Clench (amplitude) in microvolts - left masseter - pre-treatment	Oral submucous fibrosis	20	102.535	53.5130	.570
	Control	20	90.660	49.6033	
Clench (onset of muscle activity) in milliseconds - left masseter - pre-treatment	Oral submucous fibrosis	20	62.670	63.4549	.017
	Control	20	131.835	123.9895	

The Wilcoxon signed-ranks test compared the mean differences of rest amplitude, clench amplitude, and the onset of activity during clenching, before and after treatment in the right temporalis (Table 7) and left temporalis (Table 8) muscles and found it to be statistically not significant.

**Table 7:** Wilcoxon signed-ranks test comparing the pre-treatment and post-treatment mean differences of rest amplitude, clench amplitude, and the onset of activity during clenching in the right temporalis muscle

		Mean	N	Standard deviation	Asymptotic significance (two-tailed)
Pair 1	Rest (amplitude) in microvolts - right temporalis - pre-treatment	2.3550	20	1.45299	.156
	Rest (amplitude) in microvolts - right temporalis - post-treatment	1.8935	20	.98863	
Pair 2	Clench (amplitude) in microvolts - right temporalis - pre-treatment	118.9010	20	71.58737	.279
	Clench (amplitude) in microvolts - right temporalis - post-treatment	117.975	20	85.0830	
Pair 3	Clench (onset of muscle activity) in milliseconds - right temporalis - pre-treatment	48.125	20	55.8866	.396
	Clench (onset of muscle activity) in milliseconds - right temporalis - post-treatment	83.315	20	116.8445	

**Table 8:** Wilcoxon signed-ranks test comparing the pre-treatment and post-treatment mean differences of rest amplitude, clench amplitude, and the onset of activity during clenching in the left temporalis muscle

		Mean	N	Standard deviation	Asymptotic significance (two-tailed)
Pair 1	Rest (amplitude) in microvolts - left temporalis - pre-treatment	2.2015	20	1.44434	.573
	Rest (amplitude) in microvolts - left temporalis - post-treatment	1.9520	20	.82830	
Pair 2	Clench (amplitude) in microvolts - left temporalis - pre-treatment	111.305	20	52.1386	.067
	Clench (amplitude) in microvolts - left temporalis - post-treatment	99.325	20	60.4084	
Pair 3	Clench (onset of muscle activity) in milliseconds - left temporalis - pre-treatment	52.890	20	54.2847	.014
	Clench (onset of muscle activity) in milliseconds - left temporalis - post-treatment	107.945	20	124.2779	

The Wilcoxon signed-ranks test compared the pre-treatment and post-treatment mean differences of rest amplitude, clench amplitude, and the onset of activity during clenching, before and after treatment in the right masseter (Table 9) and left masseter (Table 10) muscles were evaluated. The differences in amplitude during clench in the right masseter of the OSMF patients were statistically significant with a p-value of 0.007, with a pre-treatment mean value of 133.370 µV and a post-treatment mean value of 102.775 µV. Similarly, the differences in amplitude during clench in the left masseter of the OSMF patients were statistically significant with a p-value of 0.036, with a pre-treatment mean value of 102.535 µV and a post-treatment mean value of 92.090 µV.

**Table 9:** Wilcoxon signed-ranks test comparing the pre-treatment and post-treatment mean differences of rest amplitude, clench amplitude, and the onset of activity during clenching in the right masseter muscle

		Mean	N	Standard deviation	Asymptotic significance (two-tailed)
Pair 1	Rest (amplitude) in microvolts - right masseter - pre-treatment	1.6010	20	.65912	.286
	Rest (amplitude) in microvolts - right masseter - post-treatment	1.5240	20	.93676	
Pair 2	Clench (amplitude) in microvolts - right masseter - pre-treatment	133.370	20	67.5507	.007
	Clench (amplitude) in microvolts - right masseter - post-treatment	102.775	20	59.7803	
Pair 3	Clench (onset of muscle activity) in milliseconds - right masseter - pre-treatment	54.355	20	77.4924	.396
	Clench (onset of muscle activity) in milliseconds - right masseter - post-treatment	77.4924	20	86.3501	

**Table 10:** Wilcoxon signed-ranks test comparing the pre-treatment and post-treatment mean differences of rest amplitude, clench amplitude, and the onset of activity during clenching in the left masseter muscle

		Mean	N	Standard deviation	Asymptotic significance (two-tailed)
Pair 1	Rest (amplitude) in microvolts - left masseter - pre-treatment	1.6695	20	1.10577	.446
	Rest (amplitude) in microvolts - left masseter - post-treatment	1.5125	20	1.19608	
Pair 2	Clench (amplitude) in microvolts - left masseter - pre-treatment	102.535	20	53.5130	.036
	Clench (amplitude) in microvolts - left masseter - post-treatment	92.090	20	59.2750	
Pair 3	Clench (onset of muscle activity) in milliseconds - left masseter - pre-treatment	62.670	20	63.4549	.247
	Clench (onset of muscle activity) in milliseconds - left masseter - post-treatment	78.345	20	76.2581	

**Figure 15:**
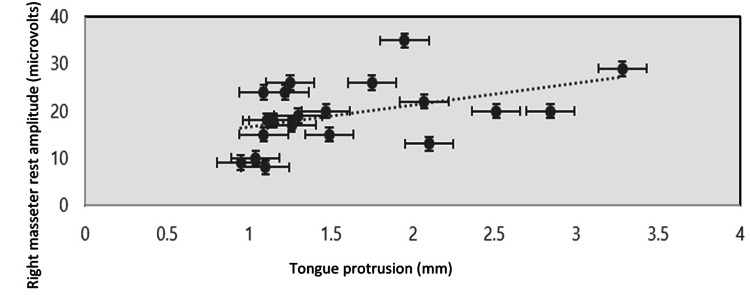
Scatter plot graphical representation of the correlation between tongue protrusion and the amplitude at rest in the right masseter muscle before treatment

The correlation analysis was done among all the pre-treatment and post-treatment assessment parameters against each other, which concluded that there exists an association between tongue protrusion and rest amplitude in the right masseter muscle in OSMF patients before treatment. The correlation coefficient (r) was 0.376 with a two-tailed statistical significance of 0.023 (Figure 15).

## Discussion

In this study, we selected 20 OSMF cases of age range 20 to 80 years without systemic comorbidities to assess pre-treatment and post-treatment changes with intralesional corticosteroid therapy as an intervention. There was no dropout of recruited cases and all patients were followed up till treatment outcome was achieved. The cases were clinically assessed based on mouth opening, tongue protrusion, mucosa colour, palpable fibrotic bands, and burning sensation. The cases were also subjected to histopathological grading by incisional biopsy of the tautest fibrotic band, which corresponded to the quit holding site. Clinical and histopathological cases were graded using Kerr et al.'s classification. As OSMF involves changes in muscle plane in moderately advanced and advanced cases, EMG was used as an assessment tool for measuring the muscle activity of the masticatory muscles, namely, temporalis and masseter, which could be recorded non-invasively with available wet gel electrodes. A total of 20 age and gender-matched individuals without systemic comorbidities were used as a healthy control for comparing the muscle action potential of masticatory muscles with OSMF patients as there are no standardized normal baseline values in masseter and temporalis muscles.

In the present study, when comparing the pre-treatment and post-treatment clinical assessment parameters in OSMF patients, there was a complete resolution of burning sensation from VAS score 8 to VAS score 0, pale blanched oral mucosa observed clinically improved to normal pink colour, and bilateral palpable fibrotic bands reduced to single unilateral palpable fibrotic band corresponding to the patient’s quid holding site. There was an increase in mouth opening and tongue protrusion from the mean value of 27.10 mm to 34.20 mm and 19.40 mm to 25.50 mm, respectively. Hence, intralesional corticosteroid therapy has been shown to improve the clinical presentation of OSMF.

Among electromyographic assessment parameters, in the masseter muscle, the mean rest amplitude of the right and left masseter reduced from 1.6010 µV to 1.5240 µV and 1.6695 µV to 1.5125 µV, respectively. The mean clench amplitude of the right and left masseter reduced from 133.370 µV to 102.775 µV and 102.535 µV to 92.090 µV, respectively. The mean onset of muscle activity during clenching in the right and left masseter increased from 54.355 ms to 68.855 ms and 62.670 ms to 78.345 ms, respectively. A linear association was seen between tongue protrusion and rest amplitude in the right masseter muscle in untreated OSMF patients (r = 0.376, p-value: 0.023).

Comparing the electromyographic values before and after therapy, the differences in amplitude during clench in the right masseter of the OSMF patients were statistically significant with a p-value of 0.007 with a pre-treatment mean value of 133.370 µV and a post-treatment mean value of 102.775 µV. Similarly, the differences in amplitude during clench in the left masseter of the OSMF patients were statistically significant with a p-value of 0.036, with a pre-treatment mean value of 102.535 µV and a post-treatment mean value of 92.090 µV.

In the right masseter, during rest, the amplitude of the OSMF group, with a mean value of 1.6010 µV was statistically significant with a p-value of 0.050 when compared with the control group, with a mean value of 4.1275 µV. During clench, the amplitude of the OSMF group, with a mean value of 133.370 µV, was statistically significant with a p-value of 0.062 when compared with the control group, with a mean value of 94.310 µV.

In the left masseter, during rest, the amplitude of the OSMF group, with a mean value of 1.6695 µV, was statistically significant with a p-value of 0.066 when compared with the control group, with a mean value of 2.5735 µV. During clench, the differences in onset of muscle action (duration) of the OSMF group, with a mean value of 62.670 ms were statistically significant with a p-value of 0.017 when compared with the control group, with a mean value of 131.835 ms.

There are very few studies that utilized EMG in OSMF. The muscle involvement in OSMF had been previously reported ultrastructural by light [15] and electron microscopic studies [16]. The connective tissue undergoes alterations with the deposition of dense collagen with a fragmented worm-like appearance. The fragmentation, nuclei internalization, multiple nuclei, and striations-less eosinophilic muscle fibres were the most common structural changes in OSMF in the muscle plane [21]. Increased duration of quid chewing habit leads to increased masseter muscle thickness due to overdemand caused by excess masticatory force. Thus masseter muscle hypertrophy is a proven parameter of OSMF that could be morphologically assessed using ultrasonography (USG) [22].

The functional assessment of masseter muscle could be measured with sEMG by recording the generated electrical activity during rest and clench movements [23,24]. EMG records myoelectrical signals by evaluating their production and quantifies them in measurable units [25]. The parameters measured in EMG are the timing of muscle activation and contractile force [26]. Muscle contraction is due to the components in the plasma membrane of the muscle fibre, namely, myofibrils [27] and sarcolemma [28]. In the presence of adenosine triphosphate (ATP), muscle fibres get shortened with calcium ions available in sarcoplasm for binding with troponin [29]. Resting EMG is thus the muscle activity in the silent period caused by ATP depletion [30]. Though various researchers have modified EMG to its current form, the pioneering role in EMG device development is attributed to Carlo Matteuci [31]. Muscle action potential represents the summation of independent discharges from the motor unit which gets disturbed in morphological changes of the muscle unit in degenerative diseases that get reflected as altered waveforms in EMG.

The surface EMG is the local measurement of total action potentials of the muscle presented within the volume detection range by placing wet gel electrodes over them [32]. The muscle activity onset and activated muscle contractile force are expressed in µV and mA, respectively [33]. Kant et al. [17] used EMG in 40 clinically diagnosed OSMF patients to assess the involvement of masseter, orbicularis oris, and temporalis by comparing with healthy comparators and found that changes in the muscle activity of the masseter muscle were statistically significant in OSMF than other muscles.

Sinha et al. [18] in their non-randomized clinical trial used EMG in 30 clinically and histopathologically graded OSMF patients to assess the masseter muscle's activity changes before and after using intralesional corticosteroid therapy as an intervention and compared them with 30 healthy comparators. They found that there was increased EMG activity in the case group before treatment when compared with the healthy controls, and after treatment, there was decreased activity when compared with pretreatment activity, thereby quantifying the prognostic outcome.

Shandilya et al. [19] used EMG in clinically diagnosed 20 Oral submucous Fibrosis patients who were split into two groups to find the efficacy of the intervention given. One group was treated with botulinum toxin type A and the other was treated with saline followed by bilateral laser fiberotomy in both groups. One month after injection, the OSMF group showed decreased EMG activity. Patil et al. [34] in their study recruited 180 clinically diagnosed OSMF patients divided into four groups based on their clinical grading and assessed the pre-treatment and post-treatment EMG activity of the masseter muscle after one month of intralesional corticosteroid therapy against 45 healthy comparators and concluded that OSMF patients showed increased masseter activity before treatment when compared with healthy controls, which decreased after treatment.

In general, clench amplitude in OSMF patients, which measures the magnitude of force contraction, increases with the thickness of the muscle due to more fibres per unit area that get activated. The duration of onset of force, measured in ms, is influenced by muscle fibre length and degenerative changes [35-38]. The reduced onset of activity in the left masseter of the OSMF group implies that as the muscle fibre length is shortened in OSMF, the onset time is lesser. This duration therefore gets increased over the course of treatment as when mouth opening increases after treatment, the muscle length also is stretched and elongated.

Our study was the only study that assessed pre-treatment and post-treatment electrical activity of both the temporalis and masseter muscles simultaneously with intralesional corticosteroid therapy. There was no statistically significant difference in the mean electrical activity of the temporalis muscles in OSMF patients before and after intervention. In the right temporalis, between OSMF and control groups, the rest amplitude in OSMF patients with the mean value of 2.3550 µV was not statistically significant with a p-value of 0.766 when compared with the same of the other group with the mean value of 2.8790 µV. Similarly, in right temporalis, the clench amplitude in OSMF patients with the mean value of 118.9010 µV was not statistically significant with a p-value of 0.433 when compared with the same of the other group with the mean value of 92.9500 µV. Also, the clench onset of activity in OSMF patients with the mean value of 48.125 ms was not statistically significant with a p-value of 0.935 when compared with the same of the other group with the mean value of 40.730 ms.

In the left temporalis, between OSMF and control groups, the rest amplitude in OSMF patients with the mean value of 2.2015 µV was not statistically significant with a p-value of 0.598 when compared with the same of the other group with the mean value of 4.1835 µV. Similarly, in left temporalis, the clench amplitude in OSMF patients with the mean value of 111.305 µV was not statistically significant with a p-value of 0.465 when compared with the same of the other group with the mean value of 99.445 µV. Also, the clench onset of activity in OSMF patients with the mean value of 52.890 ms was not statistically significant with a p-value of 0.464 when compared with the same of the other group with the mean value of 57.645 ms. Our finding in temporalis muscle was similar to a previous study by Kant et al. [17], where right and left temporalis clench amplitudes, and onset of activity in all four OSMF groups when compared to healthy controls were not statistically significant. They did not assess resting EMG and recorded only clench activities. In their study, the right temporalis clench amplitudes in OSMF patients among groups I-IV were 407.50 µV, 389.13 µV, 367.53 µV, and 407.53 µV, respectively, and in control was 414.76 µV. The left temporalis clench amplitudes in OSMF patients among groups I-IV were 377.43 µV, 364.03 µV, 389.46 µV, and 407.53 µV respectively, and in control was 423.51 µV. The right temporalis clench onset in OSMF patients among groups I-IV were 6.40 ms, 8.42 ms, 6.12 ms, and 7.80 ms, respectively, and in control was 7.64 ms. The left temporalis clench onsets in OSMF patients among groups I-IV were 6.82 ms, 7.42 ms, 6.97 ms, and 6.94 ms, respectively, and in control was 7.44 ms.

Other studies followed by Kant et al. [17] did not measure temporalis based on their findings. We included temporalis and masseter together as the Bio-EMG device used had the capability to record temporalis and masseter simultaneously in one functional movement. The results of electrical activity in the temporalis muscle might be due to the fact that though the temporalis is part of the muscles of mastication, it does not get affected in OSMF patients as much as the masseter muscles because of its morphology. The fan-shaped superficial part of the temporalis forms the superficial tendon and lateral border of the retromolar triangle by converging inferomedially. The rectangular, vertical, narrow deep part forms the deep tendon and medial border of the retromolar triangle by converging posterolaterally [39]. Because of this anatomy and its insertion into the coronoid process, the lesser surface area of muscle fibres is in direct contact with the alkaloids of areca nut products making them remote to OSMF changes.

Regarding the masseter muscle, the results of our study were similar to previous studies [17,18,34] and not similar to one study [19]. In the study by Kant et al [17], there was no statistically significant variation observed in electromyographic clench onset among all four groups of OSMF and in comparison, with the control group. However, in group 4 OSMF patients with mouth opening <20mm, there was a statistically significant variation in clench amplitude between the right masseter with the mean value of 407.66 µV when compared with the left masseter the mean value of 358.69 µV.

In Sinha et al.'s study [18], the mean clench amplitudes of the right masseter and the left masseter were 696.56 ± 152.06 and 703.97 ± 184.10, respectively, pretreatment, and 500.73 ± 171.81 and 473.87 ± 201.99, respectively, post-treatment, which was statistically significant. Also, the mean clench amplitude values of the right masseter and left masseter, viz., 696.56 ± 152.06 and 703.97 ± 184.10, respectively, pretreatment were statistically significant to control mean values of 150.23±15.65 and 149.10±13.99 µV, respectively. In the study by Patil et al. [34], the results revealed that the EMG activity of masseter muscles in OSMF patients showed increased activity when compared with healthy comparators that decreased after intralesional corticosteroid therapy. The clench duration was statistically significant by a drop of 7.38 ms to 7.30 ms and 7.50 ms to 7.43 ms in the right and left masseter, respectively, after treatment in OSMF patients with mouth opening 20-30 mm. Also, the clench amplitude was statistically significant between OSMF and control groups with the mean value of 346.23 µV in the case and 359.07 µV in the control group in the right masseter and with the mean value of 344.97 µV in the case and 359.99 µV in the control group in left masseter after treatment in OSMF patients with mouth opening less than 20 mm. 

The results of our study were different from the study by Shandilya et al. [19], where there were no statistically significant electromyographic changes in the masseter muscle before and after treatment, though a decrease in activity was noticed. This might be due to the difference in study design and methodology wherein they had used botulinum toxin type A and saline in two OSMF groups and compared them.

Our study was the only study that found a correlation between right masseter amplitude at rest and tongue protrusion in OSMF patients. Though there are no proven differences in right and left masseter thickness in normal subjects that had been verified by morphometric assessment studies using USG and MRI [39], it could be understood as the influence of quid-holding sites in right-handed individuals. As they tend to hold the quid predominantly in the right buccal vestibule, for longer hours, the right side mucosa gets affected more than the opposite side. This could be corroborated further by the fact that even after treatment with intralesional corticosteroid therapy, a single taut fibrotic band remains in the quid-held site. Hence, it is important to do excision of the single fibrotic band either by surgery or light amplification by stimulated emission of radiation (LASER) fiberotomy for better treatment outcome.

There are a few limitations in this study. We included only the population which reported to the dental hospital for treatment. Larger samples across demographics could be studied with a single EMG machine as multicentric studies to fathom the result further. Our study sample had predominantly males like all previous studies and hence gender influences need to be considered. We included only dentulous OSMF patients in our study, and EMG levels in edentulous OSMF patients also needed to be studied in future.

There do not exist any standard reference EMG values in masseter and temporalis as rest amplitude, clench amplitude, and clench onset of activity. There also exist technical manufacturing differences among the EMG devices in regard to their recording parameters. Surface electrodes were used due to non-invasiveness similar to previous studies. Needle electrodes could be used in future studies after anaesthetising the muscle area. However, patient compliance with needle electrodes is questionable. The recent re-discovery of the masseter deep third layer, masseter pars coronoidea, running from the medial surface of the zygomatic process to the coronoid process, has been shown to influence mandible action [40]. This has to be explored further using biomechanical studies to recognize its influence in the generation of muscle activity and to examine whether it could be quantified irrespective of deeper location.

Our study concluded that EMG could be used as an objective assessment tool in quantifying the prognosis of OSMF patients. Intralesional corticosteroid therapy with lycopene supplementation and physiotherapy is an effective pharmacological treatment modality in the management of OSMF. EMG values are increased in OSMF patients when compared with healthy controls before treatment and decreased after treatment due to muscle relaxation and effective balanced action.

## Conclusions

Intralesional corticosteroid therapy with lycopene supplementation and physiotherapy continues to be the effective pharmacological treatment modality in OSMF management. There was an increase in mouth opening and tongue protrusion, a reduction in palpable fibrotic bands, improvement in mucosa colour, and complete resolution of burning sensation with the given treatment modality. There was a correlation between tongue protrusion and rest amplitude in the right masseter muscle in OSMF patients before treatment. During rest, in the right and left masseter, the amplitude of the OSMF group was lower than the control group. During clench, in the right masseter, the amplitude of the OSMF group was higher than the control group. During clench in the left masseter, the onset of muscle action was lesser in the OSMF group than in the control group. After treatment, there was a reduction in clench amplitude in OSMF patients in both the right and left masseter from their pretreatment values, signifying muscle relaxation and a better onset of muscle action. Hence, EMG can be effectively used to assess the electrical activity in the masseter muscle as a prognostic tool during the management of OSMF.
